# Peripheral blood biomarkers correlate with outcomes in advanced non-small cell lung Cancer patients treated with anti-PD-1 antibodies

**DOI:** 10.1186/s40425-018-0447-2

**Published:** 2018-11-23

**Authors:** Aixa E. Soyano, Bhagirathbhai Dholaria, Julian A. Marin-Acevedo, Nancy Diehl, David Hodge, Yan Luo, Rami Manochakian, Saranya Chumsri, Alex Adjei, Keith L. Knutson, Yanyan Lou

**Affiliations:** 1 Department of Hematology and Oncology, Mayo Clinic, 4500 San Pablo Road, Jacksonville, FL 32224 USA; 2 Department of Blood and Marrow Transplantation and Cellular Immunotherapy, Moffitt Cancer Center, Tampa, FL USA; 3 Department of Internal Medicine, Mayo Clinic, Jacksonville, FL USA; 4 Department of Biomedical Statistics and Informatics, Mayo Clinic, Jacksonville, FL USA; 5 Department of Cancer Biology, Mayo Clinic, Jacksonville, FL USA; 6 Robert and Monica Jacoby Center for Breast Health, Mayo Clinic, Jacksonville, FL USA; 7 Department of Medical Oncology, Mayo Clinic, Rochester, MI USA

**Keywords:** Non-small cell lung cancer, Nivolumab, Pembrolizumab, Anti-PD-1, Immunotherapy, Relapse/progression

## Abstract

**Background:**

Anti-programmed cell death 1 (PD-1) antibodies have demonstrated improved overall survival (OS) and progression-free survival (PFS) in a subset of patients with metastatic or locally advanced non-small cell lung cancer (NSCLC). To date, no blood biomarkers have been identified in NSCLC to predict clinical outcomes of treatment with anti-PD-1 antibodies.

**Patient and methods:**

We performed an analysis of retrospectively registered data of 157 patients with advanced NSCLC treated with anti-PD-1 antibodies at Mayo Clinic in Florida and Rochester. White blood cell count, absolute neutrophil count (ANC), absolute lymphocyte count (ALC), ANC to ALC (ANC: ALC) ratio, absolute eosinophil count, absolute monocyte count (AMC), platelet counts, and myeloid to lymphoid (M:L) ratio at baseline and throughout treatment were assessed. Kaplan-Meier method and Cox proportional hazards model were performed.

**Results:**

We treated 146 patients with nivolumab and 11 with pembrolizumab between January 1, 2015 and April 15, 2017. At median follow-up of 20 months, median OS and PFS were 6.0 and 2.6 months, respectively. Higher baseline ANC, AMC, ANC: ALC ratio and M: L ratio correlated with worse clinical outcomes in patients who underwent anti-PD-1 treatment. A baseline ANC: ALC ratio of 5.9 or higher had a significantly increased risk of death (hazard ratio [HR] =1.94; 95% confidence interval [CI], 1.24–3.03; *P* = 0.004) and disease progression (HR, 1.65; 95% CI, 1.17–2.34; *P* = 0.005) compared with patients with lower ratio. Similarly, a baseline M: L ratio of 11.3 or higher had significantly increased risk of death (HR, 2.5; 95% CI, 1.54–4.05; *P* < 0.001), even after a multivariate analysis (HR, 2.31; *P* = 0.002), compared to those with lower ratio.

**Conclusions:**

Increased baseline ANC: ALC ratio and M: L ratio before initiation of anti-PD1 antibodies were associated with poor PFS and OS in advanced NSCLC patients. The potential predictive value of these readily available biomarkers might help with risk stratification and treatment strategies. These findings warrant further investigation in a larger, prospective study.

**Electronic supplementary material:**

The online version of this article (10.1186/s40425-018-0447-2) contains supplementary material, which is available to authorized users.

## Background

Lung cancer is the second most common cancer worldwide and the leading cause of cancer-related deaths among both men and women [1]. Non-small cell lung cancer (NSCLC) accounts for approximately 85% of these cases. Systemic therapy is generally indicated for patients with advanced NSCLC who present with metastatic disease or recurrence following initial definitive treatment. Combination chemotherapy with a platinum-base doublet has been the backbone of initial systemic therapy for the last decade for patients whose tumors do not have driver mutations [2]. Recently, monoclonal antibodies targeting programmed cell death protein 1 (PD-1) or its ligand (PD-L1), such as nivolumab, pembrolizumab or atezolizumab, have demonstrated improvement in overall survival (OS) and progression-free survival (PFS) in a subset of patients with metastatic or locally advanced lung cancer [3, 4]. These drugs have been approved by the Federal Drug Administration as effective options for patients with metastatic lung cancer as monotherapy or in combination with chemotherapy [5, 6].

Previous studies of patients with melanoma treated with immunotherapy targeting the cytotoxic T-lymphocyte-associated protein 4 pathway, have described hematologic parameters as predictive or prognostic markers of outcomes [7–11]. In regards to PD-1/PD-L1 pathway, a study by Weide et al. [12] in melanoma patients treated with pembrolizumab also reported certain hematologic parameters as independent predictors of favorable OS. However, very little is known regarding the value of blood biomarkers in NSCLC to predict clinical outcomes with the use of anti-PD-1 antibodies.

The aim of our study is to determine the correlation between routinely available peripheral blood biomarkers and clinical outcomes to anti-PD-1 antibodies in patients with advanced NSCLC. Specifically, we wanted to identify if any particular blood biomarker used in routine clinical practice could help predict treatment benefit or outcome and thus help with risk stratification and defining treatment selection strategies in this patient population.

## Materials and methods

### Patient population

We analyzed retrospectively-prospectively registered data from January 1, 2015 to April 15, 2017 of patients with advanced NSCLC treated with anti-PD-1 antibodies (nivolumab and pembrolizumab). Our study was approved by the Mayo Clinic Institutional Review Board. The study was conducted in accordance with the declaration of Helsinki.

### Treatment and data collection

Pembrolizumab was administered intravenously over 1 h at a dose of 2 mg/kg every 21 days and nivolumab was administered intravenously over 2 h at a dose of 3 mg/kg every 14 days per manufacturer guidelines.

The following peripheral blood cell counts were obtained at baseline and prior to each subsequent cycle of immunotherapy: white blood cell count (WBC); absolute neutrophil count (ANC); absolute lymphocyte count (ALC); absolute monocyte count (AMC); absolute eosinophil count (AEC); and platelet count. ANC to ALC (ANC:ALC) ratio and myeloid to lymphoid lineage (M:L) ratio were calculated. The M:L included a sum of myeloid cell lines (ANC + AEC + AMC) divided by ALC.

Patients’ baseline clinical and demographic characteristics and treatment-related details were collected. Clinical response to anti-PD-1 antibodies was evaluated by computed tomography of the chest, abdomen, and pelvis with or without brain magnetic resonance imaging every 8 to 12 weeks and assessed with Immune-related Response Evaluation Criteria in Solid Tumors [13]. PD-L1 status was determined by immunohistochemistry using PD-L1 22C3 antibody (Dako) according to our institutional protocol and was reported as percentage of PD-L1 staining on tumor cells.

### Statistical analysis

Patient characteristics were presented by descriptive statistics. Continuous variables were presented as median and range and categorical data as counts and percentages. For outcome analysis, PFS was defined as date of first dose of immunotherapy to date of progression on imaging or end of immunotherapy, whichever occurred first. OS was defined as date of first dose of immunotherapy to death or last follow-up. Kaplan-Meier method was used to estimate OS and PFS.

Cox proportional hazards model analysis was used to generate point estimates of hazard ratio (HR) and corresponding 95% confidence interval (CI) to estimate the risk of each individual blood biomarker with outcome. Multivariable models for death were adjusted for age at diagnosis, sex, Eastern Cooperative Oncology Group (ECOG) performance status, and number of lines of chemotherapy (0, 1, 2, ≥3); for recurrence, models were adjusted for age at diagnosis and sex. All statistical tests were 2-sided, with threshold of significance set at α = 0.05 and performed using SAS Version 9.4 (SAS Institute Inc.).

Logistic regression analysis was performed for the association between blood parameter changes from baseline to 8 weeks (δ) and OS. The optimal cutoff point for ANC:ALC ratio, AMC, and M:L ratio were assessed by the method described by Contal and O′ Quigley [14].

## Results

### Patient characteristics

Among the 180 patients with NSCLC treated with anti-PD-1 antibodies, a total of 157 patients received 2 or more treatments and were included in the study. Baseline characteristics are presented on Table 1. Nivolumab and pembrolizumab were used on 93 and 7% of the patients respectively.

**Table 1:** Baseline Characteristics

Patient Characteristics	Total (*N* = 157)
Age, median (range), y	66 (27–87)
Race, No. (%)	
White	142 (90.5)
Black or African American	7 (4.5)
Asian	3 (1.9)
Native Hawaiian/Pacific Islander	1 (0.6)
Other	4 (2.5)
Diagnosis, No. (%)	
Adenocarcinoma	108 (68.8)
Squamous	45 (28.7)
Other	4 (2.5)
Sex, No. (%)	
Female	74 (47.1)
Male	83 (52.9)
Prior chemotherapy lines, No. (%)	
0	29 (18.5)
1	78 (49.7)
2	34 (21.7)
≥3	16 (10.2)
ECOG performance status, No. (%)	
0	40 (25.5)
1	75 (47.8)
2	37 (23.5)
3	5 (3.2)
CNS disease, No. (%)	
Yes	54 (34.4)
No	103 (65.6)
Immunotherapy drug, No. (%)	
Nivolumab	146 (93.0)
Pembrolizumab	11 (7.0)
Immune adverse effects, No. (%)	
No	98 (62.4)
Yes	59 (37.6)

Median age was 66 years. Majority of patients were white (91%) compared to other races. Most patients (128 [81.5%]) received chemotherapy prior to immunotherapy treatment. PD-L1 status was reported positive when level of expression was ≥1%. PD-L1 was only assessed in 29 (18.5%) patients. Among these 29 patients, 17 (58.6%) had positive PD-L1. Median time of follow-up was 20.0 months (range 2.9–122.2). Median PFS on immunotherapy was 2.6 months (range 0.0–19.1) and median OS was 6.0 months (range, 10 days-20.4 months).

### Baseline blood biomarkers

**Fig. 1:**
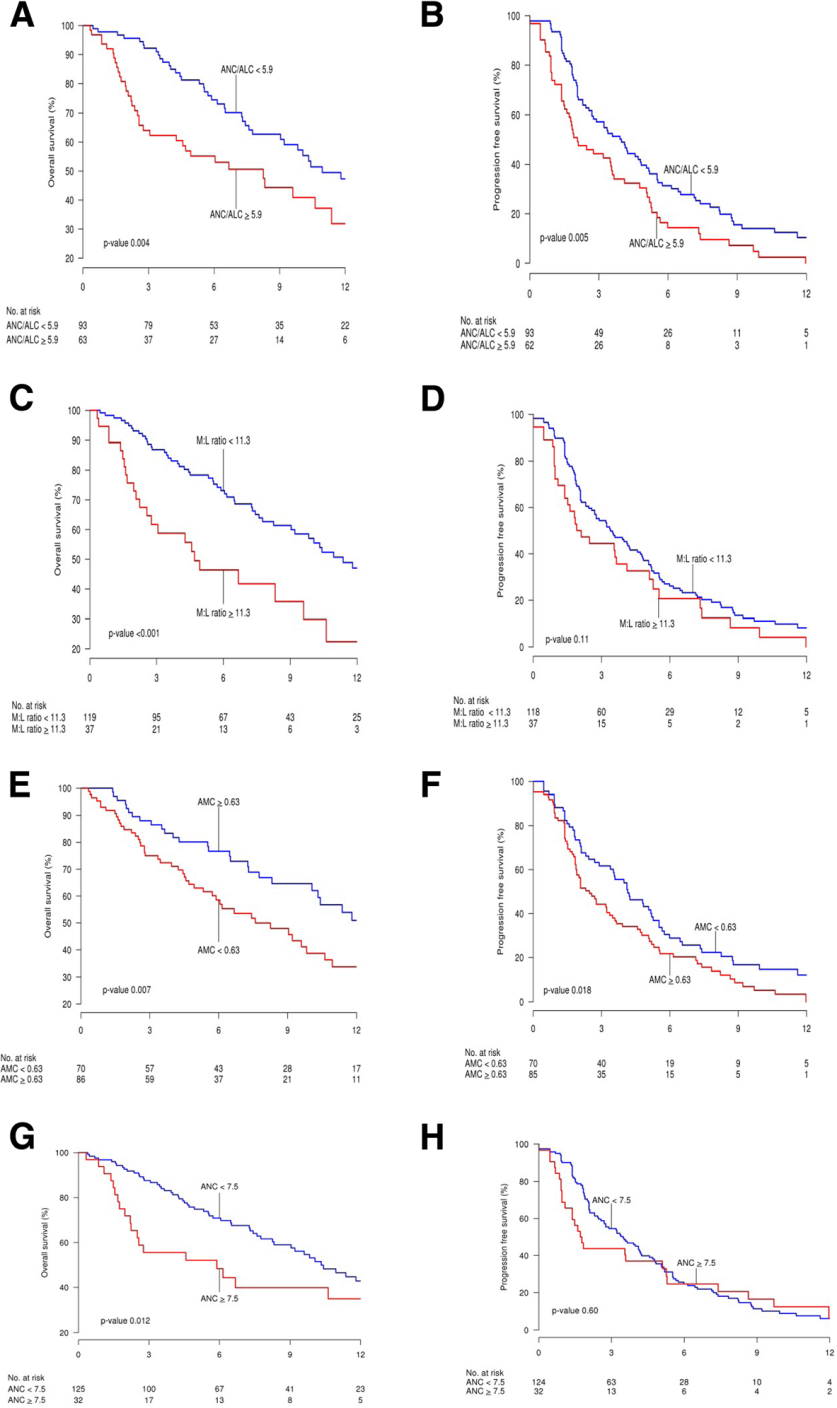
Kaplan-Meier Survival Curves for Overall Survival (OS; **a**, **c**, **e**, **g**) and Progression-Free Survival (PFS; **b**, **d**, **f**, **h**) of Non-Small Cell Lung Cancer Patients Treated With Anti-PD-1 Antibodies. Time is represented in months from start date of immunotherapy. **a** and **b**, patients are stratified by absolute neutrophil to lymphocyte (ANC:ALC) ratio. Blue lines represent ANC:ALC ratio < 5.9 and red lines, ANC:ALC ratio ≥ 5.9. **c** and **d**, patients are stratified by myeloid to lymphoid (M:L) ratio. Blue lines represent M:L ratio < 11.3 and red lines, M:L ratio ≥ 11.3. **e** and **f**, patients are stratified by absolute monocyte count (AMC). Blue lines represent AMC < 0.63 × 10^9^/L and red lines, AMC ≥ 0.63 × 10^9^/L. **g** and **h**, patients are stratified by absolute neutrophil count (ANC). Blue lines represent ANC < 7.5 × 10^9^/L and red lines, ANC ≥ 7.5 × 10^9^/L

Neutrophilia was defined as an ANC of 7.5 × 10^9^/L or higher [15]; Thirty two (20.3%) patients had neutrophilia at baseline. OS at 12 months was 34.9% (95% CI, 19.0–59.0) for patients with neutrophilia at baseline as compared to 42.9% (95% CI, 33–55.6) for patients with lower baseline ANC (*P* = 0.01) (Fig. 1). After adjusting for age, sex, ECOG performance status, and number of lines of chemotherapy, this association remained significant for death (HR, 1.86; 95% CI, 1.09–3.19; *P =* 0.02), but not progression (Table 2).

**Table 2:** Association of Baseline Blood Biomarkers and Outcomes

	Multivariate Model for PFS		Multivariate Model for OS	
Biomarker	HR (95% CI)^a^, *N = *157	*P* Value^a^	HR (95% CI)^a^, *N = *157	*P* Value^a^
WBC	1.01 (0.97–1.05)	.68	1.04 (0.99–1.09)	.10
ANC	1.01 (0.97–1.06)	.55	1.04 (1.00–1.09)	.08
ANC ≥ 7.5	1.05 (0.68–1.63)	.81	1.86 (1.09–3.19)	.02
ALC	0.78 (0.56–1.10)	.15	0.86 (0.56–1.31)	.48
ANC/ALC	1.04 (1.02–1.06)	<.001	1.04 (1.02–1.06)	<.001
ANC/ALC ≥ 5.9	1.61 (1.14–2.28)	.008	1.87 (1.16–3.02)	.01
AMC ≥ 0.63	1.50 (1.06–2.12)	.02	1.59 (0.88–2.90)	.13
AEC	0.43 (0.15–1.29)	.13	1.71 (1.06–2.75)	<.03
Platelets (per 50 unit increase)	1.03 (0.95–1.11)	.48	0.29 (0.06–1.55)	.15
M:L	1.04 (1.02–1.06)	<.001	1.05 (0.95–1.15)	.34
M:L ≥ 11.3	1.36 (0.91–2.03)	.13	1.04 (1.02–1.06)	<.001

^a^HRs, 95% CI, and *P* values result from single variable (ie, unadjusted) Cox proportional hazard models. Multivariable models were adjusted for age at diagnosis, sex, ECOG, and number of lines of chemotherapy for OS; adjusted for age at diagnosis and sex for PFS

An optimal cutoff point for AMC of 0.63 × 10^9^/L was selected based on the log rank test statistic described by Contal and O’Quigley [14]. Eighty-six patients (54.8%) had an AMC of 0.63 × 10^9^/L or higher at baseline with OS at 12 months of 33.7% (95% CI, 22.4–49.1) compared to 50.9% (95% CI, 38.3–67.8) in those with a lower baseline AMC (*P* = 0.007). A high baseline AMC was significantly associated with an increased risk of death (HR, 1.71; 95% CI, 1.06–2.75; *P* = 0.028) and progression (HR, 1.50; 95% CI, 1.06–2.12; *P* < .03) after adjusting for age, sex, ECOG performance status, and number of lines of chemotherapy.

Baseline AEC and platelet counts were not found to have significant association with response and outcomes to immunotherapy.

### ANC:ALC and M:L

An optimal cutoff point for ANC:ALC ratio of 5.9 was selected by the method of Contal and O’Quigley [14]. OS at 12 months was 31.9% (95% CI, 19.3–52.7) for patients with a baseline ANC: ALC ratio of 5.9 or higher compared to 47.3% (95% CI, 34.6–61.6) for those with a lower baseline ANC: ALC ratio (*P* = 0.004). PFS was 44.3% at 3 months and 14.4% at 6 months for patients with a higher baseline ANC: ALC ratio compared to 57.2 and 31.1%, respectively, for patients with lower baseline ANC:ALC ratio (Fig. 1). A high baseline ANC: ALC ratio was significantly associated with increased risk of death (HR, 1.94; 95% CI, 1.24–3.03; *P* = 0.004) and progression (HR, 1.65; 95% CI, 1.17–2.34; *P* = 0.005). Additional cutoff points for sensitivity analysis were assessed and can be found on the Additional file 1.

An optimal cutoff point for M:L ratio was found to be 11.3 [14]. Thirty-seven patients (23.5%) patients had a baseline M:L ratio of 11.3 or higher with OS at 12 months of 22.4% (95% CI, 9.6–52.0 compared to 47.0% (95% CI, 37.1–59.6) in patients with lower baseline M:L ratio (*P* < 0.001) (Fig. 1).A high baseline M: L ratio was significantly associated with increased risk of death (HR, 2.5; 95% CI, 1.54–4.05; *P* < .001), even after multivariate analysis (HR, 2.31; *P* = 0.002).

### Blood biomarker trends while on immunotherapy

After evaluating baseline biomarkers δ changes for values in relation to progression, we noted that an increase in WBC and ANC at 4 weeks of immunotherapy was significantly associated with higher recurrence rate after 3 months of treatment (odds ratio [OR], 1.18; 95% CI, 1.04–1.34; *P* = 0.01 for WBC, and OR, 1.20; 95% CI, 1.04–1.38; *P* = 0.01 for ANC). There was also a trend toward higher recurrence with an increase in AMC at 4 weeks (OR, 3.11; 95% CI, 0.96–10.05; *P* = 0.06) (Table 3).

**Table 3:** Association of Baseline Blood Biomarkers and δ Changes at 4 Weeks (4 Weeks – Baseline) and Recurrence after 3 Months of Immunotherapy

Biomarker	OR (95% CI) *N = *48	*P *Value
WBC	1.18 (1.04, 1.34)	0.011
ANC	1.20 (1.04, 1.38)	0.014
ALC	1.62 (0.75, 3.48)	0.22
AMC	3.11 (0.96, 10,05)	0.059
AEC (Eosinophil)	1.77 (0.42, 7.38)	0.44
Platelets	1.11 (0.90, 1.37)	0.32
ANC/ALC	1.02 (0.96, 1.09)	0.54
M:L ratio	1.02 (0.96, 1.09)	0.52

*P*-values given is based on logistic regression model

An overall increase in baseline ANC and WBC at 8 weeks after initiation of immunotherapy was statistically significant for decrease OS (HR, 1.19; 95% CI, 1.10–1.28; *P* < 0.001 for ANC, and HR, 1.18; 95% CI, 1.09–1.27; *P* < 0.001 for WBC). Increases in ANC: ALC ratio and platelet count were also significantly associated with worse OS (HR, 1.07; 95% CI, 1.04–1.11; *P* < 0.001 for ANC:ALC ratio, and HR, 1.23; 95% CI, 1.04–1.45; *P* < 0.001 for platelet count). There was no association between baseline AMC and δ change with survival (Table 4).

**Table 4:** Association of Baseline Blood Biomarkers and δ Changes at 8 Weeks with Overall Survival

	Baseline		δ at 8 Weeks	
Biomarker	HR (95% CI) *N = *157	*P *value	HR (95% CI) *N = 124*	*P *value
WBC	1.04 (1.00, 1.09)	0.043	1.18 (1.09, 1.27)	< 0.001
ANC	1.05 (1.01, 1.10)	0.029	1.19 (1.10, 1.28)	< 0.001
ALC	0.81 (0.53, 1.24)	0.33	0.95 (0.51, 1.76)	0.87
ANC:ALC	1.05 (1.03, 1.07)	< 0.001	1.07 (1.04, 1.11)	< 0.001
AMC	1.82 (1.07, 3.09)	0.027	1.45 (0.70, 3.01)	0.31
AEC	0.33 (0.07, 1.52)	0.15	1.51 (0.55, 4.10)	0.42
Platelets (per 50 unit increase)	1.08 (0.98, 1.18)	0.13	1.31 (1.12, 1.53)	< 0.001

HR, 95% CI and *P* values result from single variable (i.e. unadjusted) Cox proportional hazard models

### Safety

Immune-related adverse effects were reported in 59 patients (37.6%). There were no significant differences in the baseline demographic characteristics of patients that developed immune-related adverse events and those who didn’t. Similarly there were no significant differences in their baseline blood biomarkers (Additional file 1: Table S1).

Thyroiditis (29 [18.5%]) was the most common immune related adverse effect, followed by pneumonitis (15 [9.6%]) and rash (11 [7.0%]). Other immune adverse effects included colitis (8 [5.0%]), hepatitis (8 [5.0%]), and nephritis (7 [4.5%]). Grade 3–4 adverse effects only accounted for 4.4% of all the adverse effects. No treatment related deaths were reported. Steroid use was reported in 32 (54.2%) of the patients who developed adverse effects (Table 5).

**Table 5:** Immune related adverse effects

	*N = 157*
Immune side effects	
No	98 (62.4%)
Yes	59 (37.6%)
Pneumonitis	
Grade 1–2	13 (8.2%)
Grade ≥ 3	2 (1.3%)
Colitis	
Grade 1–2	7 (4.4%)
Grade ≥ 3	1 (0.6%)
Rash	
Grade 1–2	10 (6.3%)
Grade ≥ 3	1 (0.6%)
Thyroiditis	
Grade 1–2	27 (17.2%)
Grade ≥ 3	2 (1.3%)
Hepatitis	
Grade 1–2	7 (4.4%)
Grade ≥ 3	1 (0.6%)
Nephritis	
Grade 1–2	7 (4.4%)
Steroid use due to side effects	*N* = 59
No	27 (45.8%)
Yes	32 (54.2%)

A significantly improved OS (*P* = 0.045) was observed in patients who developed immune related adverse events and were given steroids compared to those patients that developed immune related adverse events and did not receive steroids. However, no significant association was seen with PFS in these 2 groups of patients (Additional file 1: Tables and Figures S2-S3).

## Discussion

Use of anti-PD-1 and anti-PD-L1 antibodies for treatment of multiple cancers are increasing at a fast rate, but its benefit in NSCLC seems to be limited to a subset of patients. These drugs are expensive and can cause significant immune-related adverse effects. Therefore, there is a need for reliable biomarkers to help predict response to immunotherapy. Tumor PD-L1 staining is an important predictor of response; however, it requires special immunohistochemistry testing and the optimal cutoff for positivity is debatable [16]. Tumor-infiltrating immune cells and high tumor mutation burden have recently been described as potential biomarkers of response to anti-PD-1 therapy. These are based on the fact that a higher number of neoantigens can lead to an increased activation of T cells and may enhance the antitumor immune response [17–19]. However, these tests are time consuming, experience dependent and not easily adaptable in daily clinical practice. Our study showed that readily available complete blood count data as part of routine care can help predict response to immunotherapy and clinical outcomes.

An increased ANC of 7.5 × 109/L or higher at baseline in our cohort was significantly associated with worse OS (*P* = 0.02). This finding is consistent with previous studies in melanoma using ipilimumab. Ferruci et al. [7] found that patients with ANC ≥ 7.5 × 109/L had a significantly and independently higher risk of death (HR, 3.38; 95% CI, 2.62–4.36) and progression (HR, 2.52; 95% CI, 1.97–3.21). A recent small study by Russo et al. [20] has also demonstrated that among nivolumab treated NSCLC patients, those with high baseline ANC ≥7.5 had 0% overall response rate.

In addition, we found that a baseline ANC:ALC ratio of 5.9 or higher was significantly associated with worse PFS (*P* = .008) and OS (*P* = 0.01). Zaragoza et al. [21] also found that an ANC:ALC ratio of 4 or higher in melanoma patients treated with ipilimumab was associated with worse OS in univariate and multivariate analysis, and remained as an independent prognostic factor (HR, 2.2; 95% CI, 1.01–4.78). Moschetta et al. [22] described a negative effect on PFS after 2 cycles of anti-PD-1/anti-PD-L1 in various solid tumor patients (including NSCLC) with a baseline elevated neutrophil to lymphocyte ratio of 3.4. Furthermore, our findings are similar to smaller studies in NSCLC. In a study by Diem et al. [23] they categorized ANC:ALC into 3 groups (< 3.6, 3.6–6.5 and > 6.5) and those with an elevated ANC:ALC ratio were associated with worse OS (HR, 3.64, *P* < 0.001). Naqash et al. [24], similarly to our results also found in multivariate analyses, that baseline ANC:ALC ≥5 was independently associated with inferior OS (median 5.5 vs. 8.4 months; HR 2.07, 95% CI 1.3–3.3; *P* = 0.002) and inferior PFS (median 1.9 vs. 2.8 months; HR 1.43, 95% CI 1.02–2.0; *P* = 0.04). Zer et al. [25] described an improved disease control rate (*P* = 0.025), duration of treatment (*P* = 0.037), time to progression (*P* = 0.053) and overall survival (*P* = 0.019) when patients had a low ANC:ALC ≤4 compared to a higher ANC:ALC; and there was no difference with PD-L1 expression. Furthermore, Bagley et al. [26], Labomascus et al. [27] and Preeshagul et al. [28] showed that an ANC:ALC ratio greater than 5.0 was associated with worse PFS in their respective cohorts.

Our optimal cutoff point of 5.9 for ANC:ALC was determined using the log rank statistic test described by Contal and O’Quigley [14]. In addition, an analysis of different cutoff points of 3.0 and 4.0 were also assessed in our study (Additional file 1: Tables S4-S7) and demonstrated the correlation with progression free survival, confirming the same observation. Different reference cutoff points have been used in the literature. For example, a reference of 5.0 for ANC:ALC has been used in the melanoma literature. Different cutoff points for ANL:ALC have been used in other recent NSCLC studies [22, 24, 25]. The reason for this variation is likely attributed to the baseline difference in patient population, timing of ANC:ALC in relation to the treatment and statistical methods. Future studies with larger study population are needed to further determine an optimal cutoff point. Even though the cutoff points differ between studies the conclusion stands similar; an elevated ANC:ALC at baseline in patients receiving anti-PD-1 antibodies is correlated with poor clinical outcomes such as PFS and OS.

Inflammation may enable cancer development and progression. The cytokines, interleukin 6, and tumor necrosis factor α are known to induce neutrophilia and are involved in acute inflammatory processes and in the pathogenesis of cancer-related inflammation [29, 30]. Neutrophilia, as part of the inflammatory response, can suppress the cytolytic activity of lymphocytes, activated T cells, and natural killer cells. Multiple studies have suggested an association between ANC and/or ANC:ALC ratio and the prognosis of patients with melanoma, colorectal, gastric, and renal cell cancers [31–34]. Elevations in WBC and ANC during immunotherapy treatment in our cohort were also significantly associated with higher recurrence rate after 3 months of immunotherapy (*P* = 0.01 each). Furthermore, changes in WBC, ANC, ANC:ALC ratio, and platelet count during immunotherapy were also significantly associated with worse survival (*P* < 0.001).

In our analysis, we also found that an increased M:L ratio had significant association with worse OS (*P* = 0.002). Myeloid-lineage cells may promote tumorigenesis through immunosuppression and promotion of tumor vasculature required for tumor growth and progression which could in part support and explain these findings [35]. Macrophages produce various angiogenic cytokines, including tumor necrosis factor α, interleukin 1, basic fibroblast growth factor, vascular endothelial growth factor, and transforming growth factor β, and, thus, play a key role in angiogenesis [35, 36]. Furthermore, peripheral monocytosis has also been associated with a poor prognosis in patients with lymphomas and those with solid tumors [37, 38].

A previous study suggested an increase in monocyte counts as an independent prognostic factor for poor survival in patients with metastatic melanoma treated with interleukin 2 [31]. Additionally, monocytes have been implicated as negative prognostic factors in metastatic renal cell carcinoma [39]. Consistent with these reports, we found that a high baseline AMC was significantly associated with an increased risk of death (HR, 1.71; 95% CI, 1.06–2.75; *P* < 0.03) and progression (HR, 1.50; 95% CI, 1.06–2.12; *P* = 0.02) in multivariate analyses.

Although the expression levels of PD-L1 on tumor cells and tumor-infiltrating immune cells have recently been shown to correlate with clinical response to anti-PD-1 therapy [4, 17, 40], only a subset of patients with PD-L1–expressing tumors had clinical response and others without PD-L1 staining demonstrate clinical benefit, indicating that additional factors in the tumor microenvironment exist, which define the subgroup of patients who derive benefit. Several limitations apply to PD-L1 as a predictive biomarker for immunotherapy including the dynamic changes of PD-L1 expression over time and its heterogeneity even within the same tumor. The discordance among different antibodies adds further complexity in using PD-L1 as a biomarker. Similarly, tumor mutation burden (TMB) is another surrogate biomarker. It is an indication of potential tumor antigens within the tumor. However, the definition of TMB high versus low and the optimal approach of measurement remain complex. Multiple other biomarkers such as immune gene signatures are underway. A recent study described by us [41] demonstrated a strong association between epithelial mesenchymal transition (EMT) and an inflammatory tumor microenvironment with expression of multiple immune checkpoint molecules and immune activation, indicating the potential utility of using EMT as a predictive biomarker to select patients for immune checkpoint blockade and other immunotherapies in NSCLC. However, similar to TMB, implementing a DNA or RNA-based gene signature will be clinically challenging, other simplified testing schema will need to be devised. We are currently exploring the correlation between peripheral blood biomarkers (ANL: ALC ratio and M:L ratio) and PD-L1, TMB, and EMT signature, which could be implemented in a clinical testing environment.

Our study was limited by its retrospective nature and a relatively small, predominantly Caucasian population. Potential confounders like concurrent use of medications at baseline and during treatment that could have altered the levels of the blood biomarkers, were not taken into consideration for this analysis. Most patients received nivolumab and it is unknown if these findings apply to patients treated with different anti-PD-L1 antibodies such as atezolizumab or durvalumab. Additionally, majority of patients were pretreated with chemotherapy, which may have an impact in the inflammation around the tumor, the tumor microenvironment and potentially the peripheral blood biomarkers. Despite these limitations, we believe that our study is the largest study of this nature we have found in the literature, it corroborates the findings of others and highlights the importance and potential predictive or prognostic value of these biomarkers. Other unique aspect of our analysis was exploring the role of dynamic changes in blood biomarkers during treatment and their correlation with clinical outcomes; as well as a new and important parameter the M:L ratio which can be easily incorporated into routine practice.

## Conclusions

Our data suggest that baseline ANC, AMC, and both ANC: ALC and M:L ratios prior to treatment with anti-PD-1 antibodies are associated with inferior PFS and OS in NSCLC patients. These findings might help with risk stratification and treatment strategies to avoid unnecessary toxicities and misuse of resources in patients who are less likely to benefit from treatment. ANC: ALC and M:L ratios, obtained from a complete blood count at diagnosis, are simple, widely available, and easy to use in clinical practice. In this era of precision medicine and increasing health care-associated costs, the potential predictive value of peripheral blood biomarkers for clinical outcomes with anti-PD-1 antibody treatment in lung cancer should be further investigated in a larger, prospective study.

## Additional file

Additional file 1: S1: baseline characteristics stratified by adverse events. S2-S3: Kaplan-Meier curves for OS and PFS in patients who developed immune-related adverse events. S4-S7: Kaplan-Meier curves for OS and PFS in patients who developed immune-related adverse events. S4-S7: Kaplan-Meier curves for OS and PFS with different cutoff points. (DOCX 578 kb)

## Abbreviations

AEC: Absolute eosinophil count
ALC: Absolute lymphocyte count
AMC: Absolute monocyte count
ANC: Absolute neutrophil count
CI: Confidence interval
CNS: Central nervous system
ECOG: Eastern Cooperative Oncology Group
EMT: Epithelial mesenchymal transition
HR: Hazard ratio
M: L: Myeloid to lymphoid ratio
NSCLC: Non-small cell lung cancer
OR: Odds ratio
OS: Overall survival
PD-1: Anti-programmed cell death
PD-L1: Anti-programmed cell death ligand-1
PFS: Progression-free survival
TMB: Tumor mutation burden
WBC: White blood cell
