# Supplementary material for: Peripheral blood biomarkers correlate with outcomes in advanced non-small cell lung Cancer patients treated with anti-PD-1 antibodies
Source: J Immunother Cancer. 2018 Nov 23;6:129. doi: 10.1186/s40425-018-0447-2 (PMC6251165; doi:10.1186/s40425-018-0447-2)
Supplement: Supplementary file 1 — S1: baseline characteristics stratified by adverse events. S2-S3: Kaplan-Meier curves for OS and PFS in patients who developed immune-related adverse events. S4-S7: Kaplan-Meier curves for OS and PFS in patients who developed immune-related adverse events. S4-S7: Kaplan-Meier curves for OS and PFS with different cutoff points. (DOCX 578 kb) [file 40425_2018_447_MOESM1_ESM.docx]

**Supplementary Table #1:** Baseline characteristics stratified by adverse events

| **Patients Characteristics** | **Adverse Events  (N=59)** | **No Adverse Events**  **(N=98)** | **P-Value** |
| --- | --- | --- | --- |
| **Age at diagnosis, median (range), y** | 71 (38, 87) | 64 (27, 87) | 0.11 |
| **Sex** |  |  | 0.51 |
| F | 30 (50.8%) | 44 (44.9%) |  |
| M | 29 (49.2%) | 54 (55.1%) |  |
| **Race** |  |  | 0.39 |
| White | 56 (94.9%) | 87 (88.7%) |  |
| Black or African American | 2 (3.4%) | 5 (5.2%) |  |
| Other | 1 (1.7%) | 6 (6.2%) |  |
| **Diagnosis** |  |  | 0.22 |
| Adenocarcinoma | 36 (61.0%) | 72 (73.5%) |  |
| Squamous | 21 (35.6%) | 24 (24.5%) |  |
| Other | 2 (3.4%) | 2 (2.0%) |  |
| **T**    Largest size of tumor (mm) | 38 (0, 115) | 35 (2, 150) | 0.56 |
| **N** |  |  | 0.83 |
| No | 11 (18.6%) | 16 (16.3%) |  |
| Yes | 48 (81.4%) | 82 (83.7%) |  |
| **M** |  |  | 0.093 |
| No | 29 (49.2%) | 34 (34.7%) |  |
| Yes | 30 (50.8%) | 64 (65.3%) |  |
| **History of Radiation therapy to primary tumor** |  |  | 1.00 |
| No | 28 (46.6%) | 46 (46.9%) |  |
| Yes | 31 (53.4%) | 52 (53.1%) |  |
| **CNS disease** |  |  | 0.73 |
| No | 40 (67.8%) | 63 (64.3%) |  |
| Yes | 19 (32.2%) | 35 (35.7%) |  |
| **ECOG** |  |  | 0.68 |
| 0 | 12 (21.8%) | 24 (26.4%) |  |
| 1 | 27 (49.1%) | 43 (47.3%) |  |
| 2 | 16 (29.1%) | 19 (20.9%) |  |
| 3 | 0 (0.0%) | 5 (5.5%) |  |
| **Number of lines of chemotherapy** |  |  | 0.30 |
| 0 | 16 (27.1%) | 13 (13.3%) |  |
| 1 | 24 (40.7%) | 54 (55.1%) |  |
| 2 | 13 (22.0%) | 21 (21.4%) |  |
| > 3 | 6 (10.2%) | 10 (10.2%) |  |
| **Baseline blood biomarkers, median (range)** |  |  |  |
| WBC | 6.6 (1.3, 38.0) | 7.1 (2.6, 21.5) | 0.57 |
| ANC | 4.66 (0.82, 34.50) | 4.98 (0.66, 18.51) | 0.36 |
| ALC | 1.08 (0.27, 2.99) | 0.96 (0.17, 3.43) | 0.40 |
| AMC | 0.61 (0.04, 1.71) | 0.68 (0.07, 2.27) | 0.18 |
| ANC:ALC | 4.57 (1.08, 41.00) | 5.21 (0.43, 89.00) | 0.32 |
| AEC | 0.12 (0.00, 1.77) | 0.11 (0.00, 0.91) | 0.65 |
| Platelets | 245 (72, 617) | 274 (50, 630) | 0.23 |
| M:L ratio | 5.45 (1.27, 44.38) | 6.29 (0.50, 94.00) | 0.30 |
| P-values given are based on Wilcoxson Rank Sum test or Fisher’s Exact test. | | | |

**Supplementary Table and Figure #2.** Overall Survival in patients who developed side effects based on steroid use

|  | Steroid Use No | | Steroid Use Yes | |
| --- | --- | --- | --- | --- |
| Months since diagnosis | Number at Risk | Overall Survival  (95% CI) | Number at Risk | Overall Survival  (95% CI) |
| 0 months | 27 | 100% (87.2%, 100%) | 32 | 100% (89.1%, 100%) |
| 6 months | 25 | 96.3% (89.3%, 100%) | 30 | 93.8% (85.7%, 100%) |
| 12 months | 16 | 83.7% (69.0%, 99.7%) | 23 | 74.9% (61.2%, 91.6%) |
| 18 months | 13 | 78.2% (62.2%, 97.5%) | 13 | 63.4% (46.7%, 83.6%) |
| 24 months | 8 | 72.1% (52.1%, 94.6%) | 7 | 48.8% (30.3%, 73.2%) |


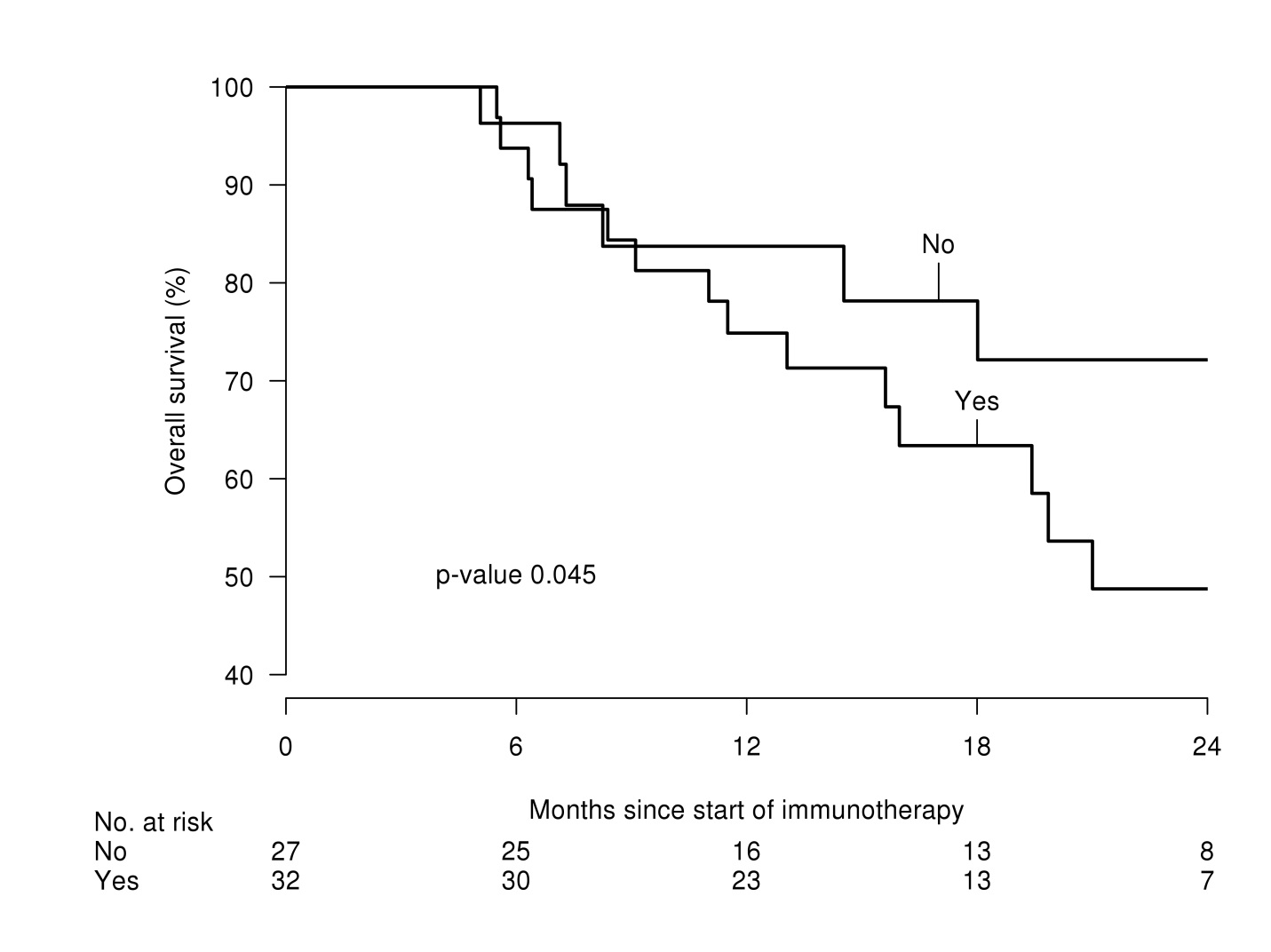


Figure #2: Kaplan-Meier Survival Curves for Overall Survival of Non-Small Cell Lung Cancer Patients Treated With Anti-PD-1 Antibodies and Developed Immune Related Side Effects Stratified by Steroid Use.

**Supplementary Table and Figure #3.** Progression free survival in patients who developed side effects based on steroid use

|  | Steroid Use No | | Steroid Use Yes | |
| --- | --- | --- | --- | --- |
| Months since immunotherapy start date | Number at Risk | Progression Free Survival  (95% CI) | Number at Risk | Progression Free Survival  (95% CI) |
| 0 months | 27 | 100% (87.2%, 100%) | 32 | 100% (89.1%, 100%) |
| 3 months | 17 | 66.7% (50.7%, 87%) | 21 | 68.6% (54.2%, 86.8%) |
| 6 months | 8 | 36.9% (21.8%, 62.3%) | 13 | 41.3% (26.9%, 63.3%) |
| 9 months | 6 | 27.6% (14.3%, 53.5%) | 1 | 4.6% (0.7%, 30.3%) |
| 12 months | 2 | 18.4% (6%, 43.9%) | 1 | 4.6% (0.7%, 30.3%) |


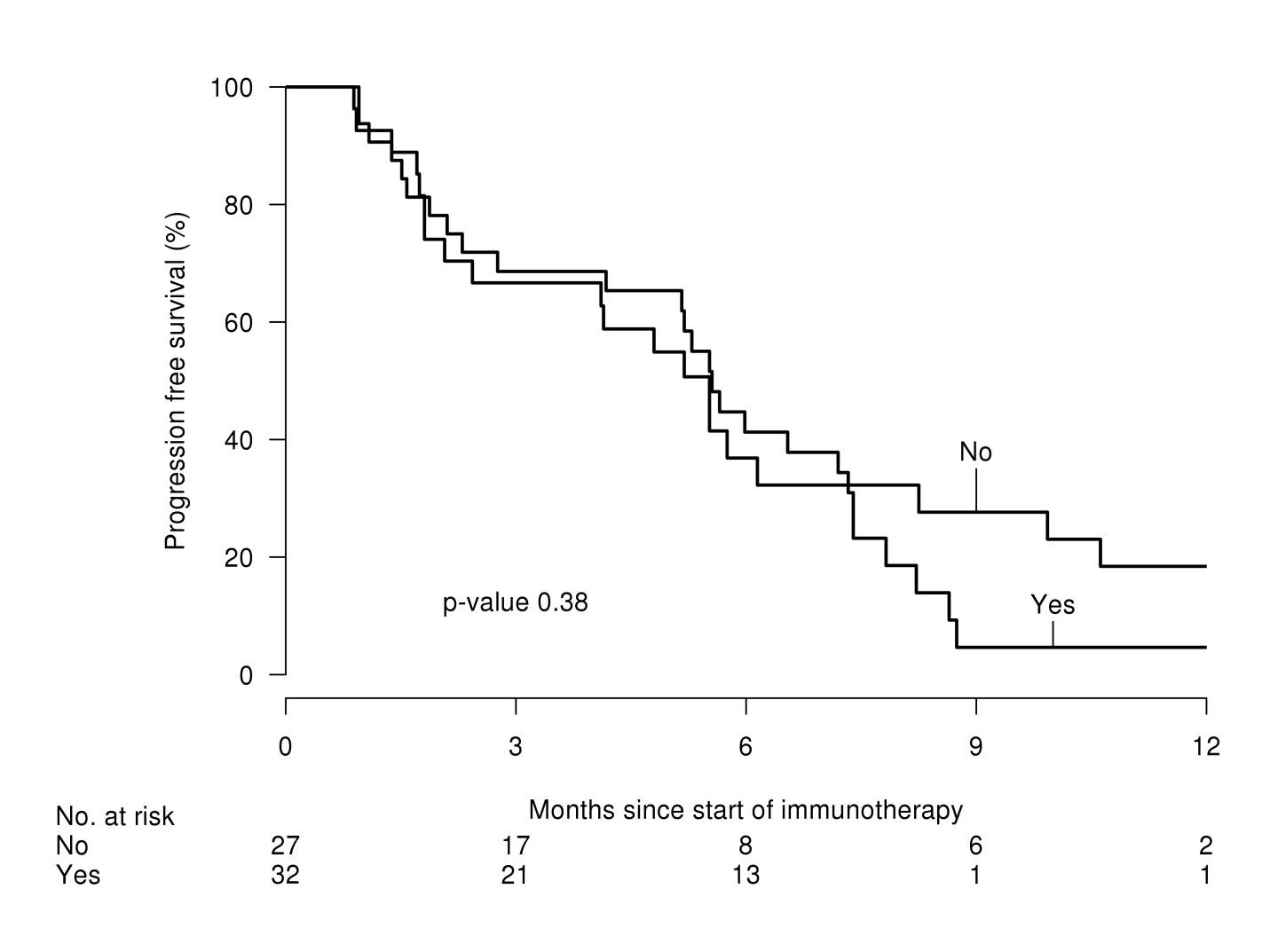


Figure #3: Kaplan-Meier Survival Curves for Progression Free Survival of Non-Small Cell Lung Cancer Patients Treated With Anti-PD-1 Antibodies and Developed Immune Related Side Effects Stratified by Steroid Use.

**Supplementary Table and Figure #4.** Overall Survival based on ANC:ALC cutoff 3.0

|  | Baseline ANC:ALC < 3.0 | | Baseline ANC:ALC > 3.0 | |
| --- | --- | --- | --- | --- |
| Months since diagnosis | Number at Risk | Overall Survival  (95% CI) | Number at Risk | Overall Survival  (95% CI) |
| 0 | 94 | 100% (96.2%, 100%) | 63 | 100% (94.3%, 100%) |
| 6 months | 86 | 94.6% (90.2%, 99.3%) | 52 | 88.6% (81%, 96.9%) |
| 12 months | 57 | 73.6% (64.8%, 83.6%) | 30 | 62.8% (51.2%, 77%) |
| 18 months | 37 | 60.6% (50.5%, 72.6%) | 21 | 53.8% (41.1%, 69.4%) |
| 24 months | 22 | 47.3% (36.5%, 61.3%) | 13 | 45.6% (32.1%, 62.5%) |


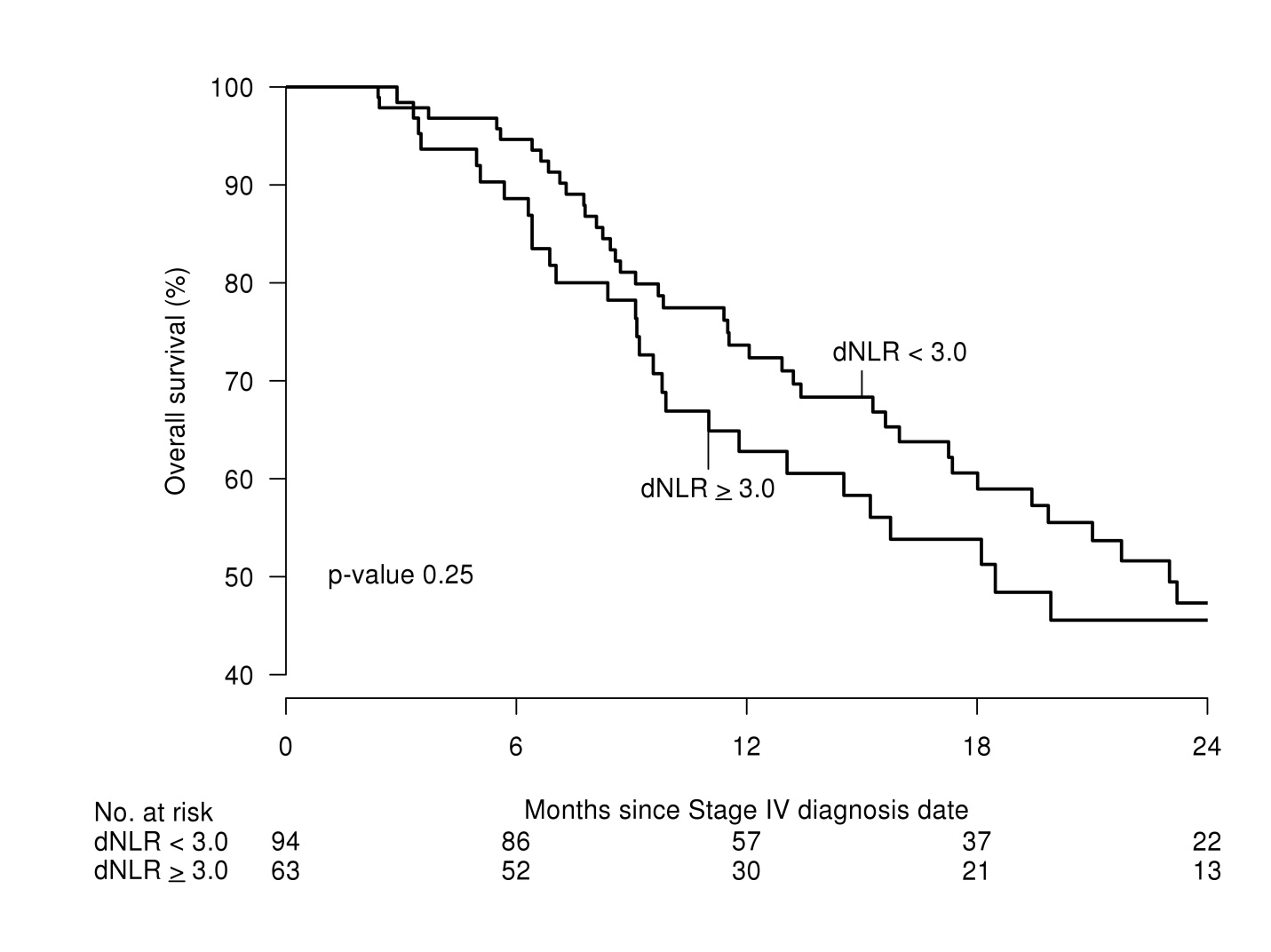


Figure #4: Kaplan-Meier Survival Curves for Overall Survival of Non-Small Cell Lung Cancer Patients Treated With Anti-PD-1 Antibodies Stratified by ANC:ALC Cutoff of 3.0

**Supplementary Table and Figure #5.** Progression Free Survival based on ANC:ALC cutoff 3.0

|  | Baseline ANC:ALC < 3.0 | | Baseline ANC:ALC > 3.0 | |
| --- | --- | --- | --- | --- |
| Months since immunotherapy start date | Number at Risk | Proportion free from recurrence  (95% CI) | Number at Risk | Progression Free Survival  (95% CI) |
| 0 months | 94 | 100% (96.2%, 100%) | 62 | 100% (94.2%, 100%) |
| 3 months | 52 | 59.8% (50.6%, 70.7%) | 24 | 41.0% (30.0%, 55.4%) |
| 6 months | 25 | 29.8% (21.6%, 41.0%) | 9 | 16.5% (9.0%, 30.1%) |
| 9 months | 10 | 14.0% (8.1%, 24.2%) | 4 | 9.6% (4.0%, 22.9%) |
| 12 months | 4 | 8.7% (4.0%, 19.0%) | 2 | 2.4% (0.4%, 16.1%) |


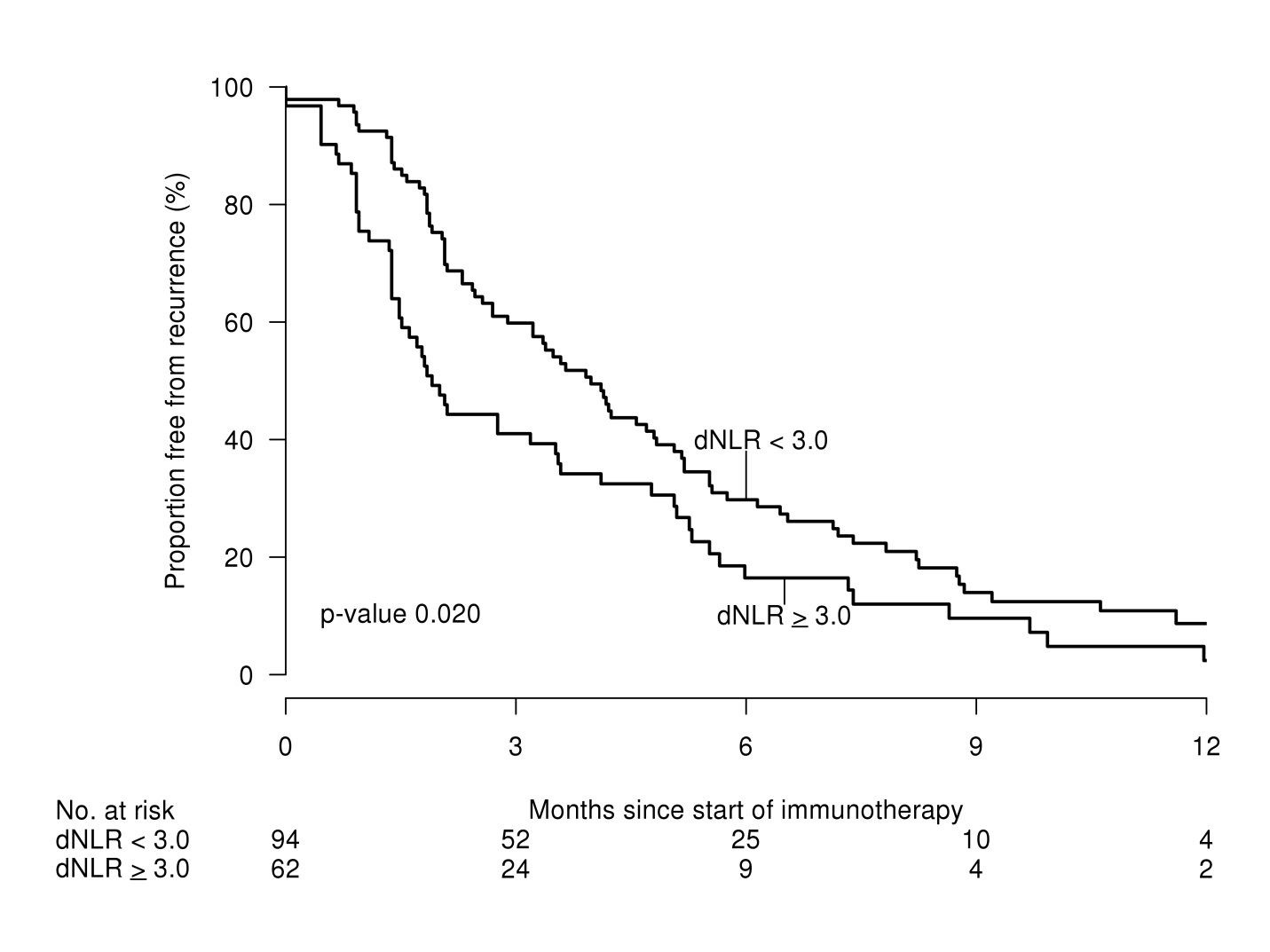


Figure #5: Kaplan-Meier Survival Curves for Progression Free Survival of Non-Small Cell Lung Cancer Patients Treated With Anti-PD-1 Antibodies Stratified by ANC:ALC Cutoff of 3.0

**Supplementary Table and Figure #6.** Overall Survival based on ANC:ALC cutoff 4.0

|  | Baseline ANC:ALC < 4.0 | | Baseline ANC:ALC > 4.0 | |
| --- | --- | --- | --- | --- |
| Months since diagnosis | Number at Risk | Overall Survival  (95% CI) | Number at Risk | Overall Survival  (95% CI) |
| 0 | 115 | 100% (96.8%, 100%) | 42 | 100% (91.6%, 100%) |
| 6 months | 100 | 92.1% (87.2%, 97.2%) | 81 | 92.8% (85.3%, 100%) |
| 12 months | 68 | 72.6% (64.4%, 81.7%) | 48 | 60.8% (46.9.5%, 78.7%) |
| 18 months | 43 | 60.6% (51.1%, 71.3%) | 30 | 50.6% (36.3%, 70.5%) |
| 24 months | 26 | 47.6% (37.7%, 60.2%) | 20 | 43.6% (27.9%, 64.5%) |


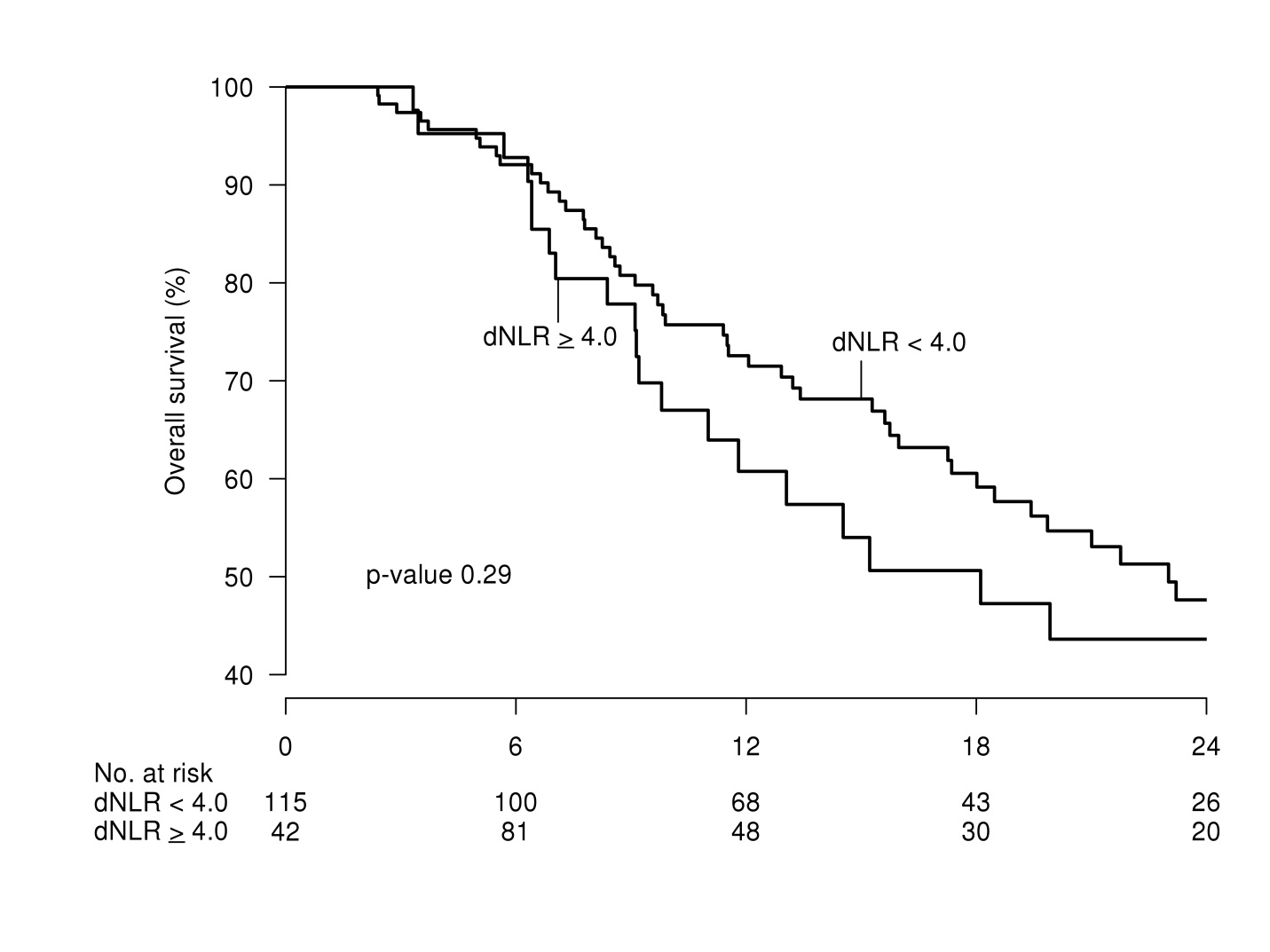


Figure #6: Kaplan-Meier Survival Curves for Overall Survival of Non-Small Cell Lung Cancer Patients Treated With Anti-PD-1 Antibodies Stratified by ANC:ALC Cutoff of 4.0

**Supplementary Table and Figure #7.** Progression Free Survival based on ANC:ALC cutoff 4.0

|  | Baseline ANC:ALC < 4.0 | | Baseline ANC:ALC > 4.0 | |
| --- | --- | --- | --- | --- |
| Months since immunotherapy start date | Number at Risk | Progression Free Survival  (95% CI) | Number at Risk | Progression Free Survival  (95% CI) |
| 0 months | 114 | 96.5% (93.2%, 99.9%) | 42 | 100% (91.6%, 100%) |
| 3 months | 59 | 55.4% (46.9%, 65.4%) | 17 | 43.9% (30.8%, 62.1%) |
| 6 months | 28 | 27.2% (19.9%, 36.9%) | 6 | 17.2% (8.2%, 36.2%) |
| 9 months | 12 | 14.0% (8.6%, 23.0%) | 2 | 6.9% (1.9%, 25.4%) |
| 12 months | 6 | 8.2% (3.9%, 17.1%) | 0 | N/A |


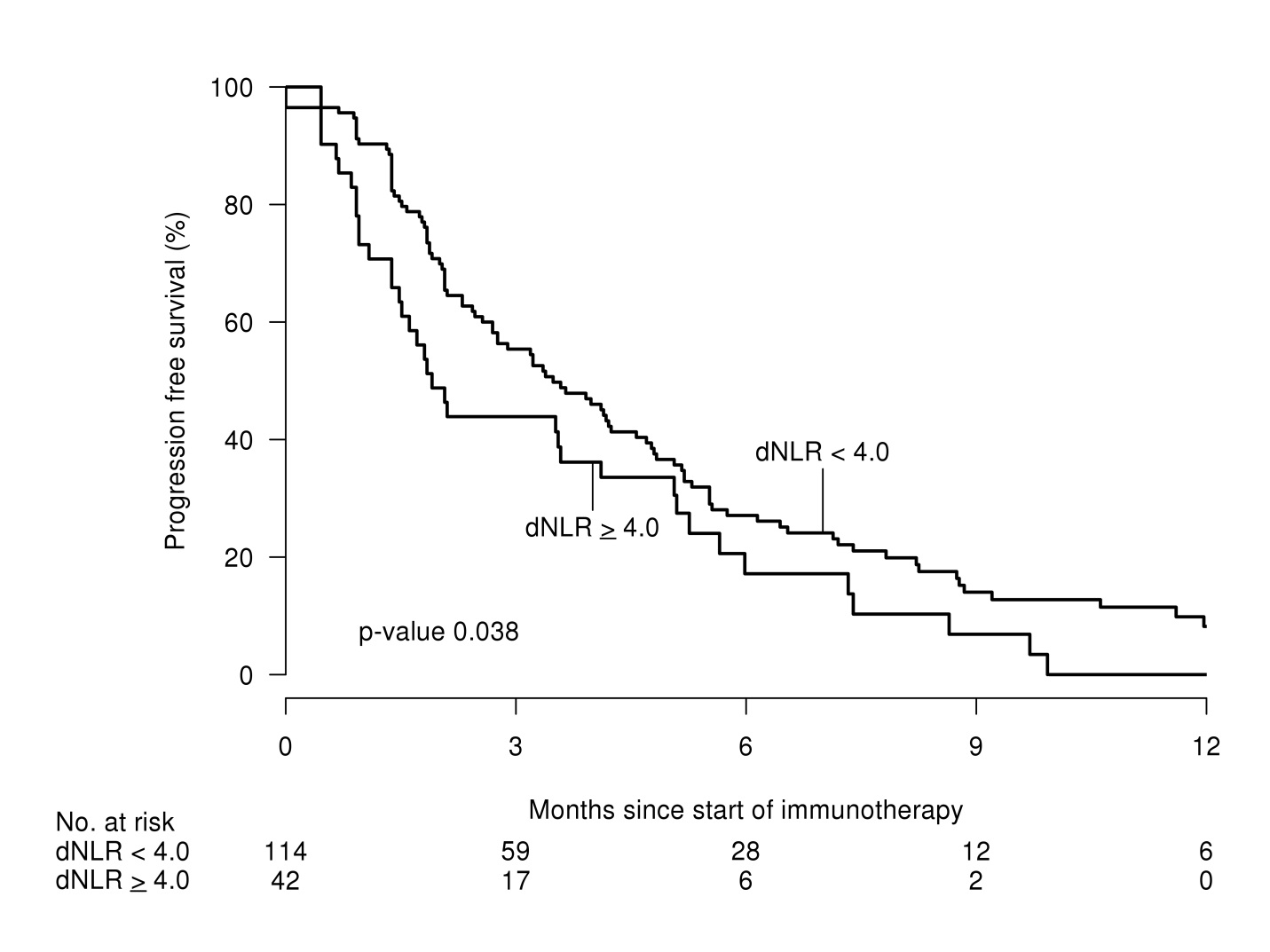


Figure #7: Kaplan-Meier Survival Curves for Progression Free Survival of Non-Small Cell Lung Cancer Patients Treated With Anti-PD-1 Antibodies Stratified by ANC:ALC Cutoff of 4.0
